# Are Autistic Traits in the General Population Stable across Development?

**DOI:** 10.1371/journal.pone.0023029

**Published:** 2011-08-04

**Authors:** Andrew J. O. Whitehouse, Martha Hickey, Angelica Ronald

**Affiliations:** 1 Telethon Institute for Child Health Research, Centre for Child Health Research, University of Western Australia, Perth, Western Australia, Australia; 2 Department of Obstetrics and Gynaecology, University of Melbourne, Melbourne, Victoria, Australia; 3 Centre for Brain and Cognitive Development, Department of Psychological Sciences, Birkbeck, University of London, London, United Kingdom; French National Centre for Scientific Research, France

## Abstract

There is accumulating evidence that autistic traits (AT) are on a continuum in the general population, with clinical autism representing the extreme end of a quantitative distribution. While the nature and severity of symptoms in clinical autism are known to persist over time, no study has examined the long-term stability of AT among typically developing toddlers. The current investigation measured AT in 360 males and 400 males from the general population close to two decades apart, using the Pervasive Developmental Disorder subscale of the Child Behavior Checklist in early childhood (M = 2.14 years; SD = 0.15), and the Autism-Spectrum Quotient in early adulthood (M = 19.50 years; SD = 0.70). Items from each scale were further divided into social (difficulties with social interaction and communication) and non-social (restricted and repetitive behaviours and interests) AT. The association between child and adult measurements of AT as well the influence of potentially confounding sociodemographic, antenatal and obstetric variables were assessed using Pearson's correlations and linear regression. For males, Total AT in early childhood were positively correlated with total AT (r = .16, p = .002) and social AT (r = .16, p = .002) in adulthood. There was also a positive correlation for males between social AT measured in early childhood and Total (r = .17, p = .001) and social AT (r = .16, p = .002) measured in adulthood. Correlations for non-social AT did not achieve significance in males. Furthermore, there was no significant longitudinal association in AT observed for males or females. Despite the constraints of using different measures and different raters at the two ages, this study found modest developmental stability of social AT from early childhood to adulthood in boys.

## Introduction

Autism is a neurodevelopmental condition characterized by impairments in social interaction and communication, and a restricted range of activities and interests [1]. Recent findings suggest that autistic traits (AT) are not limited to those with a clinical diagnosis, but can also be measured among typically developing individuals. Population-based studies have provided support for a smooth continuum of AT across the general population, with clinical autism representing the extreme end of a quantitative distribution [2]. These findings are robust, having been observed in both children and adults using a variety of assessment tools, including informant-report measures such as the Social Responsiveness Scale [3], the Quantitative Checklist for Autism in Toddlers [4], the Social and Communication Disorders Checklist [5], the Child Behaviour Checklist [6], the Communication Checklist – Adult [7], the Childhood Asperger Syndrome Test [8] and the Autism-Spectrum Screening Questionnaire [9], as well as self-report measures, such as the Autism-Spectrum Quotient [10] and the Communication Checklist – Self Report [11].

Autism is known to be a highly stable diagnosis, and there is now a substantial body of literature showing that individuals meeting criteria for autism in early childhood are highly likely to maintain this diagnosis when reassessed in adulthood [12]–[14]. Further studies have found that individual symptom profiles also remain relatively stable over time (for a review, see [15]). For example, Eaves and Ho [16] assessed 76 individuals with autism in childhood (M age = 7;6) and again in early adolescence (M age = 11;6) according to 16 diagnostic criteria (five social, six communication, and five restricted and repetitive interests). Not only did all 76 children maintain their clinical diagnosis in early adolescence, but there was a high level of concordance between time-points for the specific diagnostic criteria met (average 73%; range: 50% and 96%).

Little is known about the developmental course of AT in the general population. Constantino et al. [17] reported an intraclass correlation coefficient of 0.71 across a five year period for scores on the Social Responsiveness Scale in 95 typically developing boys who ranged in age from 3 to 18 years old. Robinson and colleagues [18] assessed 6,539 children from the Avon Longitudinal Study of Parents and Children and found no mean change in scores on the Social and Communication Disorders Checklist from ages 7 to 13 years, even in individuals with highest levels of autism-like behaviours. However, while these studies suggest developmental stability of AT when assessed at different childhood ages, no investigation has explored the longitudinal association of these behaviors from very early childhood to adulthood.

The current study investigated the association between AT measured at age two years and again at age 19–20 years among a large cohort of typically developing individuals. As well as studying total AT, the longitudinal stability of social (difficulties with social interaction and communication) and non-social (restricted and repetitive behaviours and interests) AT was assessed separately. Previous studies of clinical samples have found that developmental trajectories are not uniform across the range of autistic symptoms (e.g. [19]). Furthermore, twin studies suggest that social and non-social AT may have partly different genetic and environmental causes when assessed in the general population [8], [20]–[21], and investigations have identified multiple factor solutions underlying AT, with social and non-social AT loading onto separate factors (for reviews, see [22]–[23]). This has led to the proposal of the ‘fractionable autism triad’ hypothesis, which suggests that autism may comprise multiple underlying traits that are partly independent and should be studied separately [23]–[24].

The specific behaviours characterizing AT are likely to vary from childhood to adulthood. For example, social AT observed in young children include poor eye contact, reduced social responsiveness and conflict during peer interactions [4]. Difficulties with these more ‘fundamental’ social skills are less common as children grow older - perhaps due to the typical course of development or because of formal (e.g., therapeutic intervention) or informal (feedback from peers) instruction - and social AT in adolescence and adulthood typically manifest in other ways, such as conversational ‘bluntness’, poor topic maintenance, or a failure to read subtle social cues [25]–[26]. Cross-sectional studies of typically developing individuals have also provided evidence for age-related changes in the behaviours reflecting non-social AT [27], where, for example, restricted and repetitive interests may manifest in childhood as a strong attachment to a particular toy or video [28], but in adulthood as an intense interest in certain focused hobbies or pursuits [29]. Our hypothesis for the current study was that, while the specific behaviours of individuals would change over time, there would be developmental stability in the severity of behaviours reflecting social and non-social AT.

## Methods

### Participants

Recruitment of the Western Australian Pregnancy Cohort (Raine) Study has previously been described in detail [30]. In brief, 2900 pregnant women were recruited into a randomised controlled trial to evaluate the effects of repeated ultrasound in pregnancy. Recruitment took place at King Edward Memorial Hospital in Perth, Western Australia, or nearby private practices. The potential for introducing bias by using a tertiary referral centre population was minimised by enrolling women who booked before 18 weeks of gestation, so excluding those referred with complications. Ninety percent of eligible women agreed to participate in the study. The criteria for enrollment were a gestational age between 16 and 18 weeks, English language skills sufficient to understand the implications of participation, an expectation to deliver at KEMH, and an intention to remain in Western Australia to facilitate future follow-ups of their child(ren). From the 2900 pregnancies recruited into the Raine study, 2868 children and their families have been invited to take part in follow-ups at ages 1, 2, 3, 5, 10, 14, 16 and 20 years. Participant recruitment and all follow-ups of the study families were approved by the Human Ethics Committee at KEMH and/or Princess Margaret Hospital for Children in Perth. Informed written consent was obtained from all mothers and offspring who participated in this study.

### Autistic traits in Toddlers

At the two-year follow-up (M age = 2.14, SD = 0.16), caregivers completed the Child Behavior Checklist for Ages 1.5–5 years (CBCL) [31]. The Pervasive Developmental Problems (PDP) scale consists of 13 statements from the broader checklist. For a complete list of items in the PDP scale, refer to Edelson and Saudino [6]. Parents respond as to whether a particular statement is very true (score of 2), somewhat true (score of 1) or not true (no score), and the overall PDP index ranges from 0 (no autism traits) to 26 (high levels of autism traits). Scores from cases with missing data on up to five items were prorated to yield a score out of 26. Data were coded as missing when seven or more items were not answered. The PDP scale has 80% sensitivity for detecting children meeting criteria for autism as defined by the Autism Diagnostic Observation Scale - Generic [32]. The internal consistency for the PDP scale in the sample of Raine participants investigated in the current study was in the moderate range (Cronbach's alpha = 0.60).

The PDP scale can be further divided into two subscales, relating to social and non-social AT, as reported elsewhere [32]. From the PDP scale, six items related to social behaviours (e.g., paucity of social interaction, not answering to name) and five items to non-social behaviours (e.g., preference for sameness, repetitive body motions), with a further two PDP items excluded because they do not clearly relate to either category (“speech problems” and “strange behaviors”). Within each subscale, individual item scores were tallied to provide an overall index ranging from 0 to 12 (Social subscale) or 0 to 10 (Non-social subscale), with higher scores indicating greater severity of traits. The prorating procedure described above was applied to these scales as well. The internal consistency for the Social (Cronbach's alpha = 0.51) and Non-social subscales (Cronbach's alpha = 0.43) in the current sample was poor to modest.

### Autistic traits in early adulthood

At age 19–20 years (M age = 19.51, SD = 0.71), the cohort was invited to complete the Autism Spectrum Quotient (AQ). Adults with a known diagnosis of any developmental disorder (including autism) were not asked to take part in this study due to ethical concerns.

The AQ is a self-report questionnaire that provides a quantitative measure of AT in the general population [10]. Individuals are provided with 50 statements and asked to indicate on a 4-point scale how well that statement applied to them (strongly agree, agree, disagree, strongly disagree). The statements are divided into five subscales based on Baron-Cohen et al. [10]: Social Skills (‘I prefer to do things with others rather than on my own’), Attention to Detail (‘I am fascinated by numbers’), and Attention Switching (‘I enjoy doing things spontaneously’), Communication (‘People often tell me that I keep going on and on about the same thing’) and Imagination (‘I like to collect information about categories of things). Items within each subscale are then summed to provide a quantitative measure of that particular AT, with higher scores denoting increased symptomatology. To align AQ data with the CBCL data collected at age two years, a Social subscale was created by summing the Social Skills and Communication subscales, and a Non-social subscale was created by summing the Attention to Detail, Attention Switching and Imagination subscales. The internal reliability of the Social (Cronbach's alpha = 0.48) and Non-social subscales (0.4) was low to moderate, which is in keeping with previous analyses of AQ subscales [33]. A total AQ was calculated by tallying scores from these two subscales. The scale is known to have good test-retest (r = 0.7) and has been shown to discriminate well between high-functioning people with autism and unaffected adults [10]. Validation studies have also found that the AQ is sensitive to AT in the general population, and scores on the total AQ scale have been found to follow a quantitative distribution [10].

### Covariates

In order to determine the proportion of unique variance in adult AT attributable to early childhood AT, a range of covariates known to have an influence on behavioural outcomes were also considered [34]. These variables included family sociodemographic information measured at 18 weeks gestation (e.g., maternal and paternal age at conception, maternal education and family income), maternal smoking and alcohol intake at 34 weeks gestation, and obstetric variables (birthweight, Apgar scores 5 minutes after delivery, offspring parity) measured during the perinatal period.

### Sample attrition

The aim of each Raine Study follow-up was to assess all of those children (and their families) from the original birth cohort (n = 2868) who were contactable and willing to participate. However, as with other longitudinal studies, inevitably there were children who missed one or more of the follow-ups, or who became lost to the study as they got older. Data on both the CBCL at age two years and the AQ at age 19–20 years were available for 760 individuals from the original cohort. Participant characteristics are shown in Table 1. Independent samples t-tests found that these participants did not differ from the remainder of the birth cohort on gestational age at birth (current study participants: n = 737, M = 39.19 weeks, SD = 1.98; remainder of cohort: n = 2013, M = 39.02 weeks, SD = 2.56; t(2748) = 1.68, p = .09), birthweight (current study participants: n = 759, M = 3318.35, SD = 567.39; remainder of cohort, n = 2098, M = 3274.00, SD = 639.67; t(2855) = 1.69, p = .09) and Apgar scores 5 minutes after birth (current participants: n = 757, M = 9.00, SD = 0.81; remainder of cohort: n = 2090, M = 8.99, SD = 2.14; t(2845) = 0.18, p = .86). However chi-square analyses found that the current sample were more likely to have mothers who had completed secondary school at the time of pregnancy (current study participants: 378/760 (49.7%) of participants completed secondary school; remainder of cohort: 742/2107 (35.2%) participants completed high school; χ^2^ = 49.48, df = 1, *p*<.001), and less likely to come from families who lived below the income ‘poverty’ threshold (i.e., ≥$AUD24,000) as specified by the Australian Government at the time of recruitment (current participants: 224/741 (30.2%) of participant families below poverty threshold; remainder of cohort: 959/1963 (48.9%) of participants above poverty threshold; χ^2^ = 75.82, df = 1, *p*<.001). Therefore while the current sample was representative of the broader Raine cohort in terms of obstetric factors, they were exposed to a more socially advantageous environment.

**Table 1:** Characteristics of the participant sample.

Continuous variables	n	M (SD)	range
Maternal age at conception (years)	760	29.06 (5.46)	15.93; 41.94
Paternal age at conception (years)	487	32.10 (6.32)	17.68, 56.75
Gestational age at delivery (weeks)	737	39.19 (1.98)	24; 43
Birthweight (grams)	759	3318.35 (567.39)	750; 5185
Apgar scores 5 minutes after birth	757	9.00 (0.81)	4; 10
Age CBCL data collected (years)	760	2.14 (0.15)	1.93; 3.18
Age AQ data collected (years)	760	19.50 (0.70)	17.27; 21.53
Period between CBCL and AQ data collection (years)	760	17.36 (0.67)	15.22; 19.17

Categorical variables	N	n	%
Sex	760		
Male		360	47.4
Female		400	52.6
Maternal education at pregnancy	760		
Completed secondary school		378	49.7
Did not complete secondary school		382	50.3
Smoking in pregnancy	717		
None		592	82.6
1–10 cigarettes daily		63	8.8
11+ cigarettes daily		62	8.6
Alcohol consumption during pregnancy	717		
None		411	57.4
Once a week or less		262	36.5
Several times a week or more		44	6.1
Family income during pregnancy	741		
≥$24,000		517	69.8
<$24,000		224	30.2
Parity	760		
0		587	77.2
≥1		173	22.8

### Data preparation and statistical analyses

**Figure 1:**
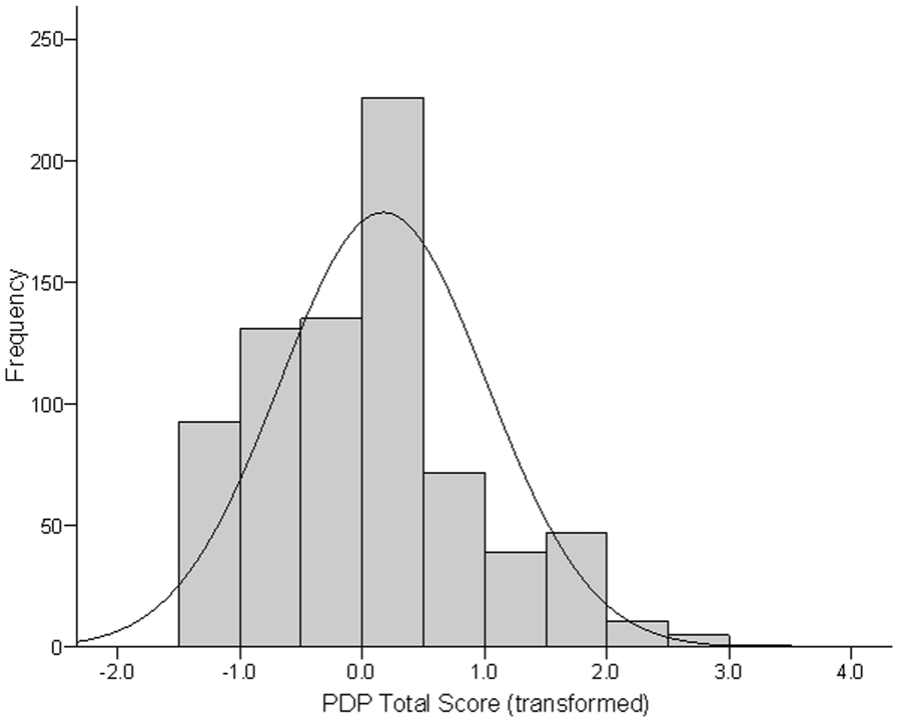
Histograms of scores on the Pervasive Developmental Disorder (PDP) scale of the Child Behaviour Checklist obtained in early childhood (transformed using cumulative frequency z-scores). Total sample size is 760 (males = 360; females = 400).

**Figure 2:**
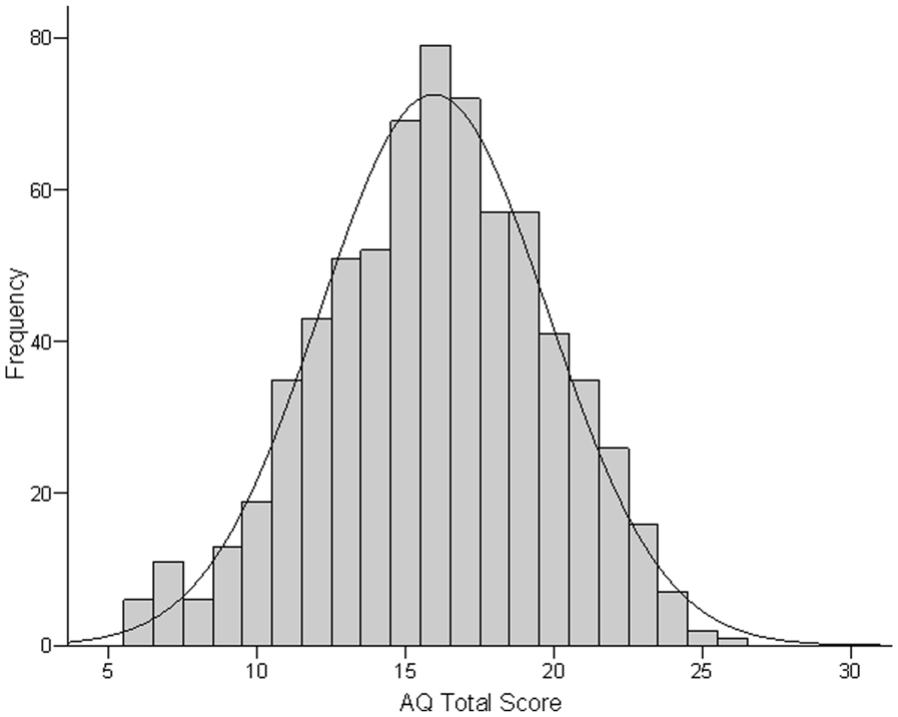
Histograms of scores on the Autism-Spectrum Quotient (AQ) obtain in early adulthood. Total sample size is 760 (males = 360; females = 400).

Prior to bivariate analyses we examined the distribution of scores on the PDP and AQ scales. The three PDP scales were found to be non-normally distributed, with skewness statistics that exceeded 1. Scores on each scale were normalised using cumulative frequency rank-transformation. Histograms of the PDP Total score (transformed) and the AQ Total score are presented in Figures 1 and 2, respectively. Pearson's correlations were then utilized to examine the correlation between the three PDP scales (Total, and the Social and Non-social subscales) and the three AQ scales (Total, and the Social and Non-social subscales) separately for each sex. A Bonferroni correction for multiple comparisons was applied so that correlations were only deemed statistically significant at the level of p<.006 (i.e., .05/9 comparisons for each sex). Significant correlations were followed-up using linear regression analyses. Any covariate that significantly correlated with the relevant outcome variable at an alpha level of p<.05, was also entered into the model. Finally, chi-square analysis examined whether toddlers scoring in the highest quintile of any of the three PDP scales, also reached this scoring threshold on the corresponding scales when assessed on early adulthood. The alpha level for these analyses was p<.05.

## Results

### PDP and AQ scores

The mean scores of the cohort on the PDP and AQ scales are shown in Table 2. The range of scores on the PDP Total (pre-transformed range: 0 to 15) and AQ Total (range: 6 to 26) scales observed in the current sample was consistent with previous investigations of the general population samples [10], [35]. Further exploratory analyses found no significant difference between males and females on any of the scales or subscales (see Table 2).

**Table 2:** Mean (SD) scores on the Pervasive Developmental Scale (PDP) of the CBCL measured at age 2 years (untransformed) and AQ and age 19–20 years (raw scores).

Scale	Subscale	Total cohort	Male	Female	P value
PDP	Total	3.06 (2.42)	3.12 (2.51)	3.00 (2.33)	0.49
	Social subscale	1.28 (1.34)	1.31 (1.34)	1.24 (1.33)	0.44
	Nonsocial subscale	1.63 (1.44)	1.65 (1.43)	1.60 (1.45)	0.68
AQ	Total	16.03 (3.84)	16.28 (3.89)	15.80 (3.81)	0.12
	Social subscale	5.12 (2.12)	5.19 (2.16)	5.08 (2.11)	0.48
	Nonsocial subscale	10.91 (2.71)	11.04 (2.73)	10.72 (2.68)	0.10

Scores are presented for the total sample (n = 698), as well separately for females (n = 400) and males (n = 360). P values are for independent t-tests comparing male and female scores (PDP comparisons with transformed scores).

To further examine the validity of the PDP scale as a measure of AT [32], we investigated the PDP scores of the children from the Raine cohort diagnosed with autism, who were not included in the broader analyses in the current study because they did not complete the AQ in adulthood (see Methods). At the 5-, 8-, 10-, 13- and 16-year follow-ups of the Raine cohort, parents were asked whether their child had ever received a diagnosis of an autism spectrum disorder (Autistic Disorder, Pervasive Developmental Disorder - Not Otherwise Specified, or Asperger Syndrome) by a health professional. Data on the PDP scale at the 2 year follow-up were available for 15 of the 16 children who had been reported to have received a diagnosis of one of these conditions. While the small number of children with an autism spectrum disorder prevented statistical analysis, nine of the 15 children with ASD had PDP Total scores greater than one standard deviation above the mean of the remainder of the cohort (i.e., ≥6), which suggests that this scale is sensitive to autistic behaviours.

The pattern of sample attrition meant that the current participants experienced a more social advantageous environment than the remainder of the Raine cohort. To determine whether this may have influenced the distribution of AT observed in the current study, we compared the PDP and AQ Total scores between those children from families who had household incomes above (n = 517) and below (n = 224) the level of qualification for government benefits (≥$AUD24,000). Chi-square analyses revealed a similar gender profile in the low income group (Males = 107, Females = 117) compared with the remainder of the sample (Males = 247, Females = 250), χ^2^ = 0.00, df = 1, *p* = 1, and therefore males and females were combined for these analyses. Independent t-tests found no statistically significant difference between the two income groups in both the PDP Total score (low income: M = 0.20, SD = 0.88; remainder of sample: M = 0.15, SD = 0.83), t(739) = 0.75, p = .46, and the AQ Total score (low income: M = 16.13, SD = 4.13; remainder of sample: M = 15.92, SD = 3.76), t(739) = .69, p = .49.

### Longitudinal AT associations

Table 3 presents correlations between the scores on the PDP and AQ scales for males and females. For males, the PDP Total scale was positively correlated with AQ Total scale (r = .16, p = .002) as well as with scores on the AQ Social subscale (r = .16, p<.001). A significant positive correlation was also observed between the PDP Social subscale and the AQ Total score (r = .17, p = .001) as well as the AQ Social subscale (r = .16, p = .002). The PDP Non-social subscale did not correlate significantly with any of the AQ scale measuring adult AT in males. For females, the strongest correlation was between scores on the PDP Total and AQ Social subscale (r = .11, p = .02), but this did not achieve statistical significance once the alpha value was adjusted for multiple comparisons (p<.006). No other correlation between PDP and AQ scales for females approached significance, and thus these data were not considered any further.

**Table 3:** Correlations between the Pervasive Developmental Scale of the CBCL measured at age 2 years and AQ and age 19–20 years in 760 individuals.

	1	2	3	4	5	6
1. PDP Total	**1**	.76*	.81*	.06	.11	−.01
2. PDP Social subscale	.80*	**1**	.31*	.02	.06	−.03
3. PDP Non-social subscale	.83*	.42*	**1**	.03	.08	−.02
4. AQ Total	.16*	.17*	.09	**1**	.73*	.84*
5. AQ Social subscale	.16*	.16*	.09	.74*	**1**	.25*
6. AQ Nonsocial subscale	.11	.11	.05	.85*	.26*	**1**

Correlations for females (n = 400) are above the diagonal, and correlations for males (n = 360) are below the diagonal.

*p<.006.

Linear regression analyses were conducted for the four significant correlations between PDP and AQ scales in males. No covariate significantly correlated with any of the AQ scales and therefore these were not included in the regression models. The PDP Total scores significantly predicted both the AQ Total (*β* = 0.16, *R* ^2^ = 0.03, p = .002) and the AQ Social (*β* = 0.16, *R* ^2^ = .03, p<.002) scores, and PDP Social scores significantly predicted AQ Total (*β* = 0.17, *R* ^2^ = .03, p = .001) and AQ Social (*β* = 0.16, *R* ^2^ = .03, p = .002) scores.

A final analysis examined whether males with high levels of AT in toddlerhood also scored highly when assessed in adulthood. High levels of AT was defined as scoring in the upper quintile of the respective distributions, which equated to scores of 5, 2 and 3 for the Total, Social and Non-social scales of the PDP (respectively), and 19, 7 and 13 for the Total, Social and Non-social scales of the AQ (respectively). The chi-square analysis for social AT was statistically significant, χ^2^ = 4.85, df = 1, p = .03; over a third (34.4%) of toddlers who scored in the upper quintile of the PDP social scale also reached this criterion on the AQ social scale (see Table 4). While there was a statistical trend for long-term stability of high levels of Total AT, χ^2^ = 3.37, df = 1, p = .07, there was no significant relationship for Non-social AT, χ^2^ = 1.57, df = 1, p = .21.

**Table 4:** Stability of high-levels (≥80^th^ centile) of Total, Social and Nonsocial AT from toddlerhood (PDP scale of the CBCL) to adulthood (Autism Quotient) among males (N = 360).

		Toddlerhood					
		Total		Social		Nonsocial	
		<80^th^ centile	≥80^th^ centile	<80^th^ centile	≥80^th^ centile	<80^th^ centile	≥80^th^ centile
Adulthood	<80^th^ centile	198 (72.8)	55 (62.5)	175 (76.4)	86 (65.6)	177 (67)	71 (74)
	≥80^th^ centile	74 (27.2)	33 (37.5)	54 (23.6)	45 (34.4)	87 (33)	25 (26)

The numbers of cases are presented in each cell with proportions in parentheses.

## Discussion

The current study is the first to investigate the long-term stability of AT among typically developing toddlers. Several significant correlations were observed in males. Total AT in early childhood were correlated with total AT (r = .16) and social AT (r = .16) in adulthood, and there was also a positive association between social AT measured in early childhood and Total (r = .17, p = .001) and social AT (r = .16, p = .002) measured in adulthood. In contrast, no significant longitudinal relationships were observed among females. These findings suggest that there is modest developmental stability of certain AT from early childhood to early adulthood among typically developing boys.

The longitudinal correlations for AT observed in the current study are comparably weaker than those reported in previous studies of other quantitative traits in toddlers, such as internalizing (r = 0.29−0.23) and externalizing (r = 0.46–0.49) behaviours [36]. It is possible that the current study may have underestimated the longitudinal association in AT for several reasons. First, baseline measurements were obtained at a relatively young age (M age = 2.15, SD = 0.16). Prospective studies of clinical autism have found that the long-term predictive validity of symptoms improves considerably from age two years to age three/four years [19], which may reflect either poor specificity of autistic symptoms in toddlers or difficulties in obtaining accurate measurements of observed behaviours.

Second, differences in the questionnaires assessing AT between time-points may have influenced the longitudinal associations. An ideal methodological design might be to utilise the same assessment at both time-points, with minor modifications for child- (time-point 1) or adult-relevant behaviours (time-point 2). Although there are now several valid assessments of AT with both child and adult report forms, including the Autism-Spectrum Quotient [10], [37], and the Communication Checklist [7], [38], there was no such measure available at the time the Raine cohort were in early childhood (1991–1993). In the current study, AT in childhood were assessed using the PDP scale of the CBCL, an informant-report measure designed to evaluate clinically-relevant ‘problem behaviours’ (reflected in the positive skew of the raw scores on the PDP scales). In comparison, the AQ, completed in adulthood, is a self-report measure that quantifies subtle AT in the general population. It is possible that differences between the two questionnaires led the current study to underestimate the association between early childhood and adult AT.

Third, different raters provided information on AT at age 2 (parent-report) and age 20 (self report), because these are the most appropriate raters for these two age groups. It has been shown that even when using the same measure at the same age, different raters provide somewhat different information. For example, parent, teacher and self reports on *the same measure* of AT (an abbreviated version of the Childhood Asperger Syndrome Test) collected *at the same time* on a representative UK sample, yielded modest correlation coefficients of between 0.2 and 0.3 [39]. Thus, in the present study, it is likely that some lack of covariance in AT will be attributable to different raters being employed across the two ages. Finally, the relatively weak correlations may reflect the poor internal reliability of both the CBCL and AQ scales. In the current study, Cronbach's alpha for the scales and subscales ranged from poor (0.4) to moderate (0.6). These modest indices may be a reflection of the relatively few number of items in each subscale, or that the division of items into Social and Non-social subscales was based on a top-down process to mirror the division of items in the DSM-IV-TR criteria for autism (as seen elsewhere; Ronald et al. [20]). However, it is noteworthy that, despite the young age of the cohort at first measurement, the use of different assessment tools and raters at the two time-points, the extensive time gap between measurements, and the poor psychometric properties of the scales, we identified several significant correlations that were suitably strong to survive a Bonferroni correction for multiple comparisons (p<.006).

The ‘continuum’ view of autism presents significant advantages to the study of the causes of the condition. Inclusion of less severely affected cases may facilitate the recruitment of larger sample sizes, which are often necessary to explore (genetic) associations with the variable autistic phenotype [3]. Longitudinal studies of clinical autism have shown that both the nature and severity of symptoms persist from early childhood to adulthood [13], [16]. The two previous longitudinal studies of AT have been confined to briefer periods of development within childhood [17]–[18]. Therefore the current study provides important new information to this research area, showing that the long-term stability of symptoms in clinical autism also extend, at least to a modest degree, to AT in the general population.

An intriguing finding from the current study was that the longitudinal AT associations were stronger for males compared to females; indeed no longitudinal association for females achieved nominal significance. It is possible that this finding reflects measurement error associated with the use of different AT scales (and raters) in early childhood and adulthood; a study design that may favour scoring for males over females, or vice versa. However, it is notable that there were no gender differences in the means (and standard deviations) of AT scores at both time points (Table 2). An alternative possibility is that AT in males and females follow a different developmental course. Although the two previous studies in this area found a high degree of stability for AT traits among both males and females across middle childhood [17]–[18], the current investigation is the first to provide a baseline measurement in early childhood. Several studies have reported that male toddlers have a more stable developmental profile compared with female toddlers, particularly with regards to problematic behaviours [40]–[42] – though, these findings have not always been replicated [43]–[44]. The identification and diagnosis of autism in toddlers has only reached acceptable levels of accuracy and reliability in the past decade [19], and therefore studies on the long-term trajectory of these children are yet to emerge. However, early reports suggest that there may be behavioural differences between male and female toddlers diagnosed with autism [45]. Longitudinal studies tracking AT from early life (in diagnosed autism as well as the general population), will provide an important examination of the current findings.

The long-term stability of social and non-social AT was investigated separately; while social AT showed modest (but significant) long-term stability in males, the current study identified no significant longitudinal associations for non-social AT. A variety of explanations can be considered. One possibility is that the non-social items of the PDP and AQ questionnaires did not measure the same underlying constructs. Previous work that has examined the factor structure of non-social autistic traits among typically developing children [28] and those with clinical autism [46]–[47] has provided evidence for at least two subtypes of behaviours: ‘repetitive sensorimotor’ and ‘an insistence on sameness’. An examination of individual PDP and AQ items reveals that both assessments predominantly measured the latter type of behaviours. For example, the Non-social subscales of the PDP and AQ both index negative feelings experienced by the disruption of a routine (PDP: “Disturbed by any change in routine”; AQ: ”It does not upset me if my daily routine is disturbed”, reverse-scored). The high degree of similarity suggests that the lack of significant correlations for non-social AT was not due to discrepancies between the two questionnaires. A second, generic explanation for the non-significant correlation between non-social AT across ages is measurement error, potential sources of which have been described above. A third explanation is that the non-significant finding is genuine and that non-social AT do not show long-term stability from toddlerhood to young adulthood. Studies of clinical autism have provided some support for this proposal. For example, several large cross-sectional studies have identified considerable variation in the severity and type of restricted and repetitive interests from childhood to adulthood [29], [48]. Longitudinal studies have elucidated this further, finding that, while repetitive sensorimotor behaviours (e.g., hand or finger mannerisms, unusual sensory interests) tend to remain stable from early to middle childhood in clinical autism, there is a significant increase in the variety of behaviours reflecting an ‘insistence on sameness’ [47]. The current study extends these findings, demonstrating, with as much certainty as can be attributed to a negative finding, that there appears to be a lack of long-term stability of non-social AT, as measured here, in typically developing toddlers.

The prospective data and large sample size are strengths of the current study. However, we acknowledge that the pattern of sample attrition means that the participants assessed in early adulthood experienced a more socially-advantageous environment than the original, unselected cohort. However, importantly, we found no evidence that social strata, indexed by household income during pregnancy, were associated with scores on the PDP or AQ. Furthermore, a recent paper from a similar prospective cohort [49] found that, while sample attrition in longitudinal cohorts are typically non-random, this attrition does not invalidate the regression models used to predict behavioural phenotypes. We therefore suggest it is unlikely that the pattern of sample attrition would have influenced the broad pattern of findings observed in the current cohort.

In conclusion, the positive associations identified in the current study indicate that there is a modest degree of stability for social AT across development in typically developing individuals. These novel findings combine with studies of individuals with a diagnosis of autism [12]–[13] in suggesting that AT have a degree of developmental stability across the whole population. This study presents unique longitudinal data and is therefore of considerable value to the field. However, it is important to highlight that the methodological limitations described previously may have led to an underestimation of the magnitude of association. Future research in this area will build upon the findings presented here, leading to greater insights into the variation of AT in the general population as well as understanding into disease processes at the clinical extreme.

## References

[1] American Psychiatric Association (2000). Diagnostic and Statistical Manual of Mental Disorders, Fourth Edition, Text Revision (DSM-IV-TR).

[2] Plomin R, Haworth CMA, Davis OSP (2009). Common disorders are quantitative traits.. Nat Rev Genet.

[3] Constantino JN, Todd RD (2003). Autistic traits in the general population: A twin study.. Arch Gen Psych.

[4] Allison C, Baron-Cohen S, Wheelwright S, Charman T, Richler J (2008). The Q-CHAT (Quantitative CHecklist for Autism in Toddlers): A normally distributed quantitative measure of autistic traits at 18–24 months of age: Preliminary report.. J Aut Dev Disord.

[5] Skuse DH, Mandy WPL, Scourfield J (2005). Measuring autistic traits: heritability, reliability and validity of the Social and Communication Disorders Checklist.. The Brit J Psychiat.

[6] Edelson L, Saudino K (2009). Genetic and environmental influences on autistic-like behaviors in 2-year-old twins.. Behav Genet.

[7] Whitehouse AJO, Bishop DVM (2009). Communication Checklist - Adult (CC-A).

[8] Ronald A, Happé F, Bolton P, Butcher LM, Price TS (2006). Genetic heterogeneity between the three components of the autism spectrum: A twin study.. J Am Acad Child Psy.

[9] Posserud MB, Lundervold AJ, Gillberg C (2006). Autistic features in a total population of 7–9-year-old children assessed by the ASSQ (Autism Spectrum Screening Questionnaire).. J Child Psychol Psych.

[10] Baron-Cohen S, Wheelwright S, Skinner R, Martin J, Clubley E (2001). The Autism-Spectrum Quotient (AQ): Evidence from Asperger Syndrome/high-functioning autism, males and females, scientists and mathematicians.. J Aut Dev Disord.

[11] Bishop DVM, Whitehouse AJO, Sharp M (2009). Communication Checklist - Self Report (CC-SR).

[12] Billstedt E, Carina Gillberg I, Gillberg C (2007). Autism in adults: symptom patterns and early childhood predictors. Use of the DISCO in a community sample followed from childhood.. J Child Psychol Psych.

[13] Howlin P, Goode S, Hutton J, Rutter M (2004). Adult outcome for children with autism.. J Child Psychol Psych.

[14] Whitehouse AJO, Watt HJ, Line EA, Bishop DVM (2009). Adult psychosocial outcomes of children with specific language impairment, pragmatic language impairment and autism.. Int J Lang Comm Dis.

[15] Matson J, Horovitz M (2010). Stability of autism spectrum disorders symptoms over time.. J Dev Phys Disab.

[16] Eaves LC, Ho HH (1996). Brief report: Stability and change in cognitive and behavioral characteristics of autism through childhood.. J Aut Dev Disord.

[17] Constantino JN, Abbacchi AM, Lavesser PD, Reed H, Givens L (2009). Developmental course of autistic social impairment in males.. Dev Psychopathol.

[18] Robinson EB, Munir K, Munafo MR, Hughes M, McCormick MC (2011). Stability of autistic traits in the general population: Further evidence for a continuum of impairment.. J Am Acad Child Psy.

[19] Charman T, Taylor E, Drew A, Cockerill H, Brown JA (2005). Outcome at 7 years of children diagnosed with autism at age 2: predictive validity of assessments conducted at 2 and 3 years of age and pattern of symptom change over time.. J Child Psychol Psych.

[20] Ronald A, Happé F, Plomin R (2005). The genetic relationship between individual differences in social and nonsocial behaviours characteristic of autism.. Dev Sci.

[21] Ronald A, Larsson H, Anckarsäter H, Lichtenstein P (in press). A Swedish twin study of autism symptoms.. Mol Psych.

[22] Mandy WP, Skuse DH (2008). Research Review: What is the association between the social-communication element of autism and repetitive interests, behaviours and activities?. J Child Psychol Psych.

[23] Happé F, Ronald A (2008). The ‘fractionable autism triad’: A review of evidence from behavioural, genetic, cognitive and neural research.. Neuropsych Rev.

[24] Happé F, Ronald A, Plomin R (2006). Time to give up on a single explanation for autism.. Nat Neurosci.

[25] Hoekstra RA, Bartels M, Verweij CJH, Boomsma DI (2007). Heritability of autistic traits in the general population.. Arch Ped Adol Med.

[26] Whitehouse AJO, Coon H, Miller J, Salisbury B, Bishop DVM (2010). Narrowing the broader autism phenotype: A study using the Communication Checklist - Adult (CC-A).. Autism.

[27] Honey E, Leekam S, Turner M, McConachie H (2007). Repetitive behaviour and play in typically developing children and children with autism spectrum disorders.. J Aut Dev Disord.

[28] Leekam S, Tandos J, McConachie H, Meins E, Parkinson K (2007). Repetitive behaviours in typically developing 2-year-olds.. J Child Psychol Psych.

[29] Esbensen A, Seltzer M, Lam K, Bodfish J (2009). Age-related differences in restricted repetitive behaviors in autism spectrum disorders.. J Aut Dev Disord.

[30] Newnham JP, Evans SF, Michael CA, Stanley FJ, Landau LI (1993). Effects of frequent ultrasound during pregnancy: A randomized controlled trial.. Lancet.

[31] Achenbach TM, Edelbrock C, Howell CT (1987). Empirically based assessment of the behavioral emotional-problems of 2-year-old and 3-year-old children.. J Abnormal Child Psychol.

[32] Sikora DM, Hall TA, Hartley SL, Gerrard-Morris AE, Cagle S (2008). Does parent report of behavior differ across ADOS-G classifications: Analysis of scores from the CBCL and GARS.. J Aut Dev Disord.

[33] Austin EJ (2005). Personality correlates of the broader autism phenotype as assessed by the Autism Spectrum Quotient (AQ).. Person Indiv Diff.

[34] Whitehouse AJO, Robinson M, Zubrick SR, Ang QW, Stanley FJ (2010). Maternal life events during pregnancy and offspring language ability in middle childhood: The Western Australian Pregnancy Cohort Study.. Early Hum Dev.

[35] Ronald A, Edelson L, Asherson P, Saudino K (2010). Exploring the relationship between autistic-like traits and ADHD behaviors in early childhood: Findings from a community twin study of 2-year-olds.. J Abnorm Child Psych.

[36] Bartels M, van den Oord EJCG, Hudziak JJ, Rietveld MJH, van Beijsterveldt CEM (2004). Genetic and environmental mechanisms underlying stability and change in problem behaviors at ages 3, 7, 10, and 12.. Dev Psychol.

[37] Auyeung B, Baron-Cohen S, Wheelwright S, Allison C (2008). The Autism Spectrum Quotient: Children's version (AQ-Child).. J Aut Dev Disord.

[38] Bishop DVM (2003). Children's Communication Checklist - 2 (CCC-2).

[39] Ronald A, Happé F, Plomin R (2008). A twin study investigating the genetic and environmental aetiologies of parent, teacher and child ratings of autistic-like traits and their overlap.. Eur Child Adoles Psy.

[40] Campbell SB (1997). Behavior problems in preschool children: Developmental and family issues.. Adv Clin Child Psych.

[41] Smith CL, Calkins SD, Keane SP, Anastopoulos AD, Shelton TL (2004). Predicting stability and change in toddler behavior problems: Contributions of maternal behavior and child gender.. Dev Psych.

[42] Tremblay RE, Nagin DS, Seguin JR, Zoccolillo M, Zelazo PD, Boivin M (2004). Physical aggression during early childhood: Trajectories and predictors.. Pediatrics.

[43] Mesman J, Stoel R, Bakermans-Kranenburg M, van Ijzendoorn M, Juffer F (2009). Predicting growth curves of early childhood externalizing problems: Differential susceptibility of children with difficult temperament.. J Abnorm Child Psych.

[44] Spieker SJ, Larson NC, Lewis SM, Keller TE, Gilchrist L (1999). Developmental trajectories of disruptive behavior problems in preschool children of adolescent mothers.. Child Dev.

[45] Hartley S, Sikora D (2009). Sex differences in autism spectrum disorder: An examination of developmental functioning, autistic symptoms, and coexisting behavior problems in toddlers.. J Aut Dev Disord.

[46] Cuccaro ML, Shao Y, Grubber J, Slifer M, Wolpert CM (2003). Factor analysis of restricted and repetitive behaviors in autism using the Autism Diagnostic Interview - Revised.. Child Psych Hum Dev.

[47] Richler J, Huerta M, Bishop SL, Lord C (2007). Developmental trajectories of restricted and repetitive behaviors and interests in children with autism spectrum disorders.. Dev Psychopathol.

[48] Jacobson J, Ackerman L (1990). Differences in adaptive functioning among people with autism or mental retardation.. J Aut Dev Disord.

[49] Wolke D, Waylen A, Samara M, Steer C, Goodman R (2009). Selective drop-out in longitudinal studies and non-biased prediction of behaviour disorders.. Brit J Psychiat.

